# Levamisole Ameliorates Rheumatoid Arthritis by Downregulating the PI3K/Akt Pathway in SD Rats

**DOI:** 10.3390/ph17111504

**Published:** 2024-11-08

**Authors:** Mu Guo, Xiangbin Yu, Zesheng Yang, Hanlu Zheng, Jiahui Zhang, Junxiang Wang, Yiqi Liao, Weirui Huang, Zhaolong Lin, Yingxue Yan, Nengfu Qiu, Jianmin Chen, Yue Yu

**Affiliations:** 1 School of Pharmacy, Fujian Medical University, Fuzhou 350122, China; guomu727@163.com (M.G.); yxb4666@fjmu.edu.cn (X.Y.); 19959565916@163.com (Z.Y.); zhenghanlu0129@163.com (H.Z.); barolxicarrel@163.com (J.Z.); 18846834706@163.com (J.W.); 3240006161@student.must.edu.mo (W.H.); 18659626193@163.com (Z.L.); 2220320379@fjmu.edu.cn (Y.Y.); qiunengfu123@163.com (N.Q.); 2 Fujian Center for New Drug Safety Evaluation, Fujian Medical University, Fuzhou 350122, China; 3 Key Laboratory of Pharmaceutical Analysis and Laboratory Medicine, School of Pharmacy and Medical Technology, Putian University, Putian 351100, China

**Keywords:** levamisole, rheumatoid arthritis, adjuvant-induced arthritis, PI3K/Akt signaling pathway, Sprague–Dawley rat

## Abstract

**Background/Objectives:** Rheumatoid arthritis (RA) is a systemic chronic autoimmune disease characterized by a protracted course, high rates of morbidity, and disability yet lacks effective therapeutic modalities. Levamisole (LVM), an immunomodulatory drug, has been clinically reported for its potential in RA treatment, while its therapeutic mechanism toward RA remains to be elucidated. Hence, this study provides theoretical support for the application of LVM in the treatment of RA. **Methods:** This study employed male Sprague–Dawley (SD) rats to construct the adjuvant-induced arthritis (AIA) model, administering LVM orally (5 mg/kg, 15 mg/kg, and 45 mg/kg) for 25 days. An evaluation of LVM’s therapeutic effects on RA was conducted through arthritis index scores, paw pad thickness, paw volume, hematoxylin and eosin (H&E) staining, 3D microcomputed tomography (micro-CT) scans, serum levels of pro-/anti-inflammatory cytokines, and serum biochemical indicators. Western blotting and immunohistochemistry staining were utilized to measure the expression levels of phosphatidylinositol 3-kinase/protein kinase B (PI3K/Akt) proteins in synovial and ankle joint tissues. **Results:** Treatment with the median dose of LVM (15 mg/kg, M-LVM) significantly reduced the arthritis index (*p* < 0.01), paw pad thickness (*p* < 0.001), and paw volume (*p* < 0.01) without affecting body weight. Additionally, M-LVM alleviated inflammatory lesions in the synovium and ankle joints and also normalized serum levels of interleukin-1 beta (IL-1β), tumor necrosis factor-alpha (TNF-α), and transforming growth factor-beta (TGF-β). The Model group exhibited significant increases in serum levels of alkaline phosphatase (ALP) (*p* < 0.01), creatine kinase (CK) (*p* < 0.05), and glucose (GLU) (*p* < 0.001) compared with the Control group; however, M-LVM effectively regulated these parameters to normal levels. Western blotting and immunohistochemistry staining revealed that PI3K-/Akt-related proteins were highly expressed in the synovial and ankle joint tissues of rats in the Model group, while treatment with M-LVM significantly reduced the expression of these proteins. Furthermore, histological examination of major organs (heart, liver, lungs, kidneys, and thymus) showed no significant pathological changes, with the exception of the spleen, where M-LVM ameliorated splenic lesions. **Conclusions:** We demonstrate that LVM at an optimal dose substantially relieves synovitis and bone erosion in AIA rats by inhibiting the PI3K/Akt signaling pathway.

## 1. Introduction

**Figure 1:**
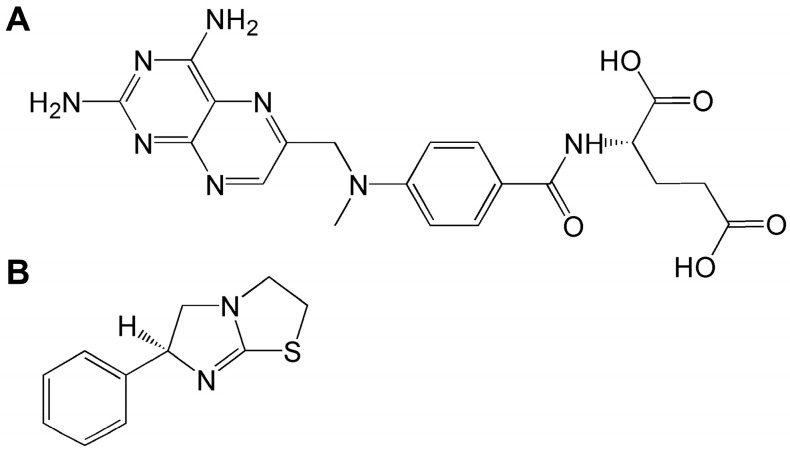
The chemical structures of (**A**) MTX and (**B**) LVM.

Rheumatoid arthritis (RA) is a chronic systemic autoimmune disease that causes systemic inflammatory responses. Studies show that the global prevalence of RA has increased by 121% since 1990, affecting approximately 18 million people in 2020 [1]. The incidence of RA varies by region and gender, with higher rates among females. The disease mainly affects joints and synovial membranes, resulting in joint swelling, pain, stiffness, and functional impairment, which can lead to arthritis and joint damage [2,3,4]. The main drugs used clinically include non-steroidal anti-inflammatory drugs, glucocorticoids, disease-modifying anti-rheumatic drugs, and biologic drugs [5]. However, the use of these drugs is limited and long-term use may result in adverse reactions, such as immunosuppression, hepatorenal dysfunction, osteoporosis, and gastrointestinal ulcers [6]. Methotrexate (MTX), a folic acid analog and antagonist (Figure 1A), was initially employed in the treatment of various malignancies but has since become a first-line therapeutic agent for RA due to its advantageous anti-inflammatory and immunomodulatory properties [7,8]. Nonetheless, the drug’s toxicity remains a significant concern, with potential adverse reactions serving as the primary rationale for treatment discontinuation [9,10].

Imidazothiazole derivatives are becoming increasingly important in biomedicine due to their excellent physiological activities. In recent years, researchers have synthesized a series of derivatives with various biological activities such as anti-tumor, anti-inflammatory, analgesic, antibacterial, and antioxidant, based on imidazothiazole as the core [11]. Levamisole (LVM), with an imidazothiazole ring as its basic structure (Figure 1B), originally developed in the mid-1960s to treat parasitic infections in animals [12], and further research has shown that LVM could be used as an adjuvant therapy for lung cancer, breast cancer following surgery, and acute leukemia and malignant lymphoma following chemotherapy and has also shown efficacy in diseases such as RA and systemic lupus erythematosus [13]. It is important to note that human studies have reported complications associated with LVM, including leukoencephalopathy, neutropenia, agranulocytosis, and skin necrosis [14,15,16]. Additionally, LVM can be metabolized into compounds with psychostimulant properties and a prolonged half-life, contributing to its widespread use alongside cocaine [17]. However, our previous research on the toxicology and pharmacokinetics of LVM in beagle dogs demonstrated that oral gavage administration of LVM for four weeks did not result in significant alterations in major organ function or serum levels, indicating a favorable safety profile for LVM [18]. Nevertheless, the potential applications of this drug in disease treatment warrant further investigation. LVM functions by regulating the body’s immunity, enhancing immunity through promoting the differentiation and proliferation of T cells, inducing the secretion of interleukin-2 (IL-2), and enhancing the phagocytic and chemotactic activities of macrophages, as well as increasing the activity of natural killer cells [19,20]. In addition, LVM could exert anti-inflammatory activity by regulating the release of tumor necrosis factor-alpha (TNF-α) and interleukin-6 (IL-6) cytokines and by increasing the activity of glutathione reductase to enhance antioxidant capacity [21,22,23,24]. In addition, reports have shown that LVM could not only inhibit the increased activity of B cells via reducing the levels of immunoglobulins and circulating immune complexes [25], but also regulate the vascular endothelial growth factor (VEGF) signaling pathway to inhibit angiogenesis [26]. Although LVM has shown therapeutic activity in several diseases, the molecular mechanism of its use in autoimmune diseases is not well understood.

Studies indicate that the pathogenesis of RA closely involves various signaling pathways, including mitogen-activated protein kinase (MAPK), phosphatidylinositol 3-kinase/protein kinase B (PI3K/Akt), and Notch [27]. The PI3K/Akt signaling pathway plays a particularly significant role in the pathophysiological processes of RA. As a classic inflammatory pathway [28,29], it regulates the production and signal transduction of several inflammatory factors, including interleukin-1β (IL-1β), IL-6, and TNF-α, which may directly participate in the occurrence of arthritis and the persistent inflammatory response [30,31]. Abnormal activation of this pathway can promote the proliferation of synovial cells and chondrocytes, exacerbating the migration and invasion of synovial and inflammatory cells, leading to synovial hyperplasia and sustained joint damage. Pathological angiogenesis aggravates pain and damage to joints and surrounding tissues, promoting systemic inflammatory responses [32,33,34,35]. Abnormal activation of the PI3K/Akt signaling pathway stimulates the expression of VEGF and hypoxia-inducible factor-1α (HIF-1α), promoting angiogenesis [36,37]. The PI3K/Akt signaling pathway can affect the activation, proliferation, and differentiation of lymphocytes, as well as the activation and chemotaxis of innate immune cells such as macrophages, dendritic cells, and neutrophils, contributing to excessive production of inflammatory cytokines and the persistence of inflammatory reactions [38,39,40]. Hence, our research group hypothesized that regulating the PI3K/Akt signaling pathway could effectively achieve RA treatment.

Therefore, finding an effective, low-toxicity treatment for RA remains a tough challenge. This study aims to clarify the therapeutic efficacy of LVM by evaluating arthritis indicators such as arthritis index scores, paw pad thickness, and paw volume, along with examining PI3K-/Akt-related protein expression in synovial and ankle joint tissues. Our research seeks to provide theoretical support for the use of LVM in RA treatment.

## 2. Results

### 2.1. LVM Suppressed RA Progress in AIA Rat Model

**Figure 2:**
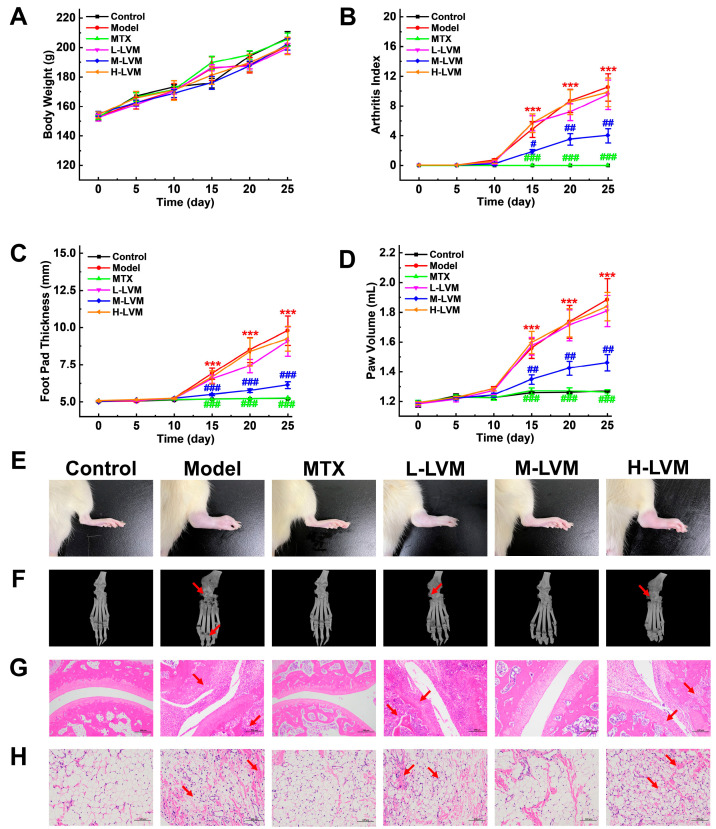
LVM alleviated RA symptoms in AIA rat model in a dose-dependent manner. (**A**) The body weight, (**B**) arthritis index, (**C**) foot pad thickness, and (**D**) paw volume were statistically analyzed. (**E**) Direct view of swelling in the right hind foot of rats. (**F**) Representative three-dimensional reconstruction images of rats’ paws from different treated groups in micro-CT. (**G**) Histological images depicting the H&E staining of ankle joint tissues (scale bar, 100 μm). (**H**) Histological images depicting the H&E staining of synovial membrane (scale bar, 100 μm). The red arrows indicate changes in the organizational structure. All data are shown as mean ± SEM. *** *p* < 0.001 when comparing with the Control group using an unpaired Student’s *t*-test; ^#^ *p* < 0.05, ^##^ *p* < 0.01, and ^###^ *p* < 0.001 when comparing with the Model group using one-way ANOVA followed by Dunnett’s post hoc test. Definitions of abbreviations for each dosing group: Control, blank control group; Model, model group; MTX, positive drug group; L-LVM, 5 mg/kg LVM group; M-LVM, 15 mg/kg LVM group; H-LVM, 45 mg/kg LVM group. *n* = 6 in each group.

The body weights of the rats in each group showed a trend of smoothly increasing, with no significant differences observed among these groups (Figure 2A). However, all the measurement results of arthritis index scores (Figure 2B), foot pad thickness (Figure 2C), and paw volume (Figure 2D) obviously demonstrate that a median dosage of LVM (M-LVM, 15 mg/kg) ameliorated foot pad swelling, as also evidenced in the direct observation of representative right hind paws in each group after sacrifice, while a low dosage of LVM (L-LVM, 5 mg/kg) and a high dosage of LVM (H-LVM, 45 mg/kg) could not exert amelioration (Figure 2E). As shown in Figure 2F, a significant reduction in bone erosion, inflammatory cell infiltration, and synovial hyperplasia after M-LVM treatment could be directly observed, which was further confirmed by the 3D microcomputed tomography (micro-CT) imaging results (Figure 2G). As demonstrated by hematoxylin and eosin (H&E) staining (Figure 2H), it was indicated that following treatment with M-LVM, a significant amelioration in the pathological status of rat synovial tissues was evident. Overall, M-LVM demonstrated therapeutic efficacy, while neither L-LVM nor H-LVM alleviated RA symptoms. As expected, MTX, a frontline clinical medication, showed the anticipated therapeutic effect in the adjuvant-induced arthritis (AIA) model.

### 2.2. LVM Regulated the Serum Levels of Pro-/Anti-Inflammatory Cytokines in AIA Rat Model

**Figure 3:**
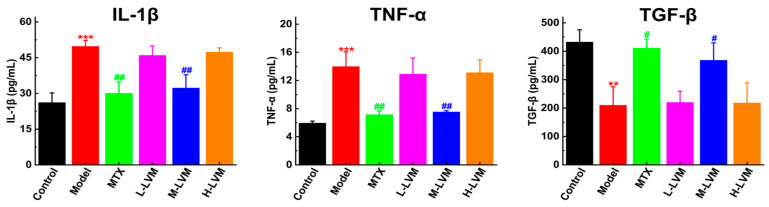
The levels of IL-1β, TNF-α, and TGF-β in the serum were measured by enzyme-linked immunosorbent assay (ELISA). All data are shown as mean ± SEM. ** *p* < 0.01, *** *p* < 0.001 when comparing with the Control group using an unpaired Student’s *t*-test; ^#^ *p* < 0.05, ^##^ *p* < 0.01 when comparing with the Model group using one-way ANOVA followed by Dunnett’s post hoc test. Definitions of abbreviations for each dosing group: Control, blank control group; Model, model group; MTX, positive drug group; L-LVM, 5 mg/kg LVM group; M-LVM, 15 mg/kg LVM group; H-LVM, 45 mg/kg LVM group. *n* = 6 in each group.

IL-1β and TNF-α, as pro-inflammatory cytokines, play a promoting role in the occurrence and progression of RA. In contrast, transforming growth factor-beta (TGF-β) exerts anti-inflammatory effects, alleviating inflammation and inhibiting disease progression. As shown in Figure 3, the levels of IL-1β and TNF-α cytokines in the serum of AIA rats were markedly elevated (*p* < 0.001), while the levels of TGF-β cytokine were significantly reduced (*p* < 0.01). Compared with the Model group, the levels of IL-1β and TNF-α cytokines in the serum of rats in the M-LVM group were significantly decreased (*p* < 0.01), and the level of TGF-β cytokine was significantly increased (*p* < 0.05). However, there were no significant differences in the levels of IL-1β, TNF-α, and TGF-β cytokines among the L-LVM, H-LVM, and Model groups.

### 2.3. LVM Normalized the Serum Biochemical Markers in AIA Rat Model

**Figure 4:**
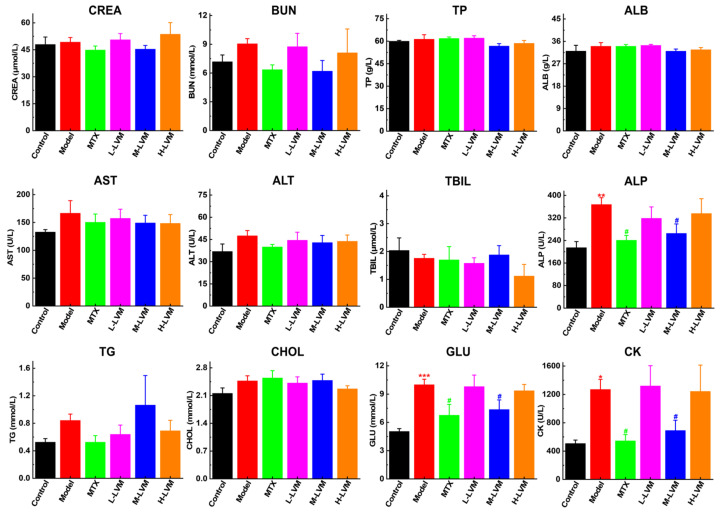
The levels of serum biochemical markers were measured by automatic biochemical analyzer. All data are shown as mean ± SEM. * *p* < 0.05, ** *p* < 0.01, and *** *p* < 0.001 when comparing with the Control group using an unpaired Student’s *t*-test; ^#^ *p* < 0.05 when comparing with the Model group using one-way ANOVA followed by Dunnett’s post hoc test. Definitions of abbreviations for each dosing group: Control, blank control group; Model, model group; MTX, positive drug group; L-LVM, 5 mg/kg LVM group; M-LVM, 15 mg/kg LVM group; H-LVM, 45 mg/kg LVM group. *n* = 6 in each group.

As shown in Figure 4, there were no significant differences observed in indicators closely associated with liver and kidney functions, such as creatinine (CREA), blood urea nitrogen (BUN), total protein (TP), albumin (ALB), aspartate aminotransferase (AST) and alanine aminotransferase (ALT), total bilirubin (TBIL), triglyceride (TG), and cholesterol (CHOL). However, anomalies were detected in the levels of alkaline phosphatase (ALP), creatine kinase (CK), and glucose (GLU). Elevated ALP is associated with impairment in hepatic, renal, and skeletal functions, while increased CK levels suggest the presence of muscle damage or muscular diseases. The findings reveal a modest elevation in ALP levels (*p* < 0.01) and CK levels (*p* < 0.05) in the Model group when compared to the Control group. In contrast, both parameters exhibited a decrease in the M-LVM group. Additionally, GLU levels were significantly increased (*p* < 0.001) in the Model group relative to the Control group, whereas a reduction in GLU levels was noted in both the MTX and M-LVM groups.

### 2.4. LVM Inhibited PI3K/Akt Signaling Pathway in Ankle Joint

**Figure 5:**
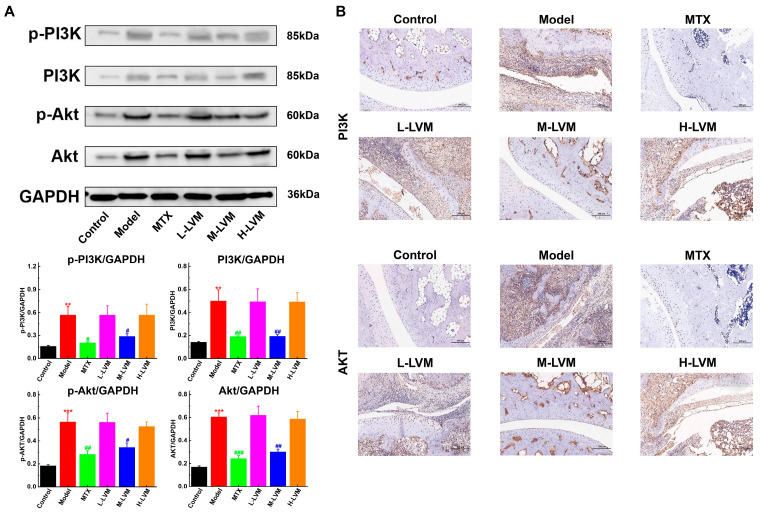
M-LVM inhibited PI3k/Akt pathway in ankle joint tissues of AIA rats. (**A**) The protein levels of p-PI3K, PI3K, p-Akt, and Akt in ankle joint tissues were detected by Western blotting. (**B**) The protein levels of PI3K and Akt in ankle joint tissues were analyzed by immunostaining (scale bar, 100 μm). All data are shown as mean ± SEM. ** *p* < 0.01, *** *p* < 0.001 when comparing with the Control group using an unpaired Student’s *t*-test; ^#^ *p* < 0.05, ^##^ *p* < 0.01, and ^###^ *p* < 0.001 when comparing with the Model group using one-way ANOVA followed by Dunnett’s post hoc test. Definitions of abbreviations for each dosing group: Control, blank control group; Model, model group; MTX, positive drug group; L-LVM, 5 mg/kg LVM group; M-LVM, 15 mg/kg LVM group; H-LVM, 45 mg/kg LVM group. *n* = 3 in each group.

As depicted in Figure 5A, compared with the Control group, the Model group exhibited significantly elevated protein expression levels of the p-PI3K (*p* < 0.01), PI3K (*p* < 0.01), p-Akt (*p* < 0.001), and Akt (*p* < 0.001) proteins. In contrast, compared with the Model group, the M-LVM group showed considerable differences in the expression of the p-PI3K (*p* < 0.05), PI3K (*p* < 0.01), p-Akt (*p* < 0.05), and Akt (*p* < 0.01) proteins, while these protein expressions in the L-LVM and H-LVM groups did not reach statistical significance.

Immunohistochemistry staining results (Figure 5B) indicate a significant increase in PI3K and Akt protein expression in the Model group compared with the Control group, along with evident cartilage erosion and fibroblast proliferation. Following M-LVM treatment, a remarkable reduction in PI3K and Akt protein expression was observed, along with alleviation of pathological symptoms, whereas the expression of the PI3K and Akt proteins and the pathological symptoms in the ankle joints of the L-LVM and H-LVM groups were similar to those in the Model group.

### 2.5. LVM Inhibited PI3K/Akt Signaling Pathway in Synovium

**Figure 6:**
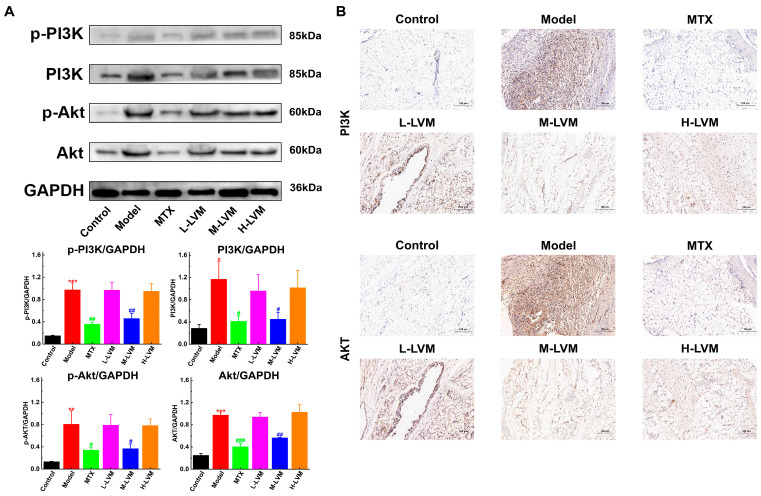
M-LVM inhibited PI3k/Akt pathway in synovial tissues of AIA rats. (**A**) The protein levels of p-PI3K, PI3K, p-Akt, and Akt in synovial tissues were detected by Western blotting. (**B**) The protein levels of PI3K and Akt in synovial tissues were analyzed by immunostaining (scale bar, 100 μm). All data are shown as mean ± SEM. * *p* < 0.05, ** *p* < 0.01, and *** *p* < 0.001 when comparing with the Control group using an unpaired Student’s *t*-test; ^#^ *p* < 0.05, ^##^ *p* < 0.01, and ^###^ *p* < 0.001 when comparing with the Model group using one-way ANOVA followed by Dunnett’s post hoc test. Definitions of abbreviations for each dosing group: Control, blank control group; Model, model group; MTX, positive drug group; L-LVM, 5 mg/kg LVM group; M-LVM, 15 mg/kg LVM group; H-LVM, 45 mg/kg LVM group. *n* = 3 in each group.

The expression levels of PI3K-/Akt-related proteins in the synovial tissues of each group were examined, as depicted in Figure 6A. Compared with the Control group, the levels of the p-PI3K (*p* < 0.001), PI3K (*p* < 0.05), p-Akt (*p* < 0.01), and Akt (*p* < 0.001) proteins were significantly increased in the Model group. Conversely, in the M-LVM group, the expression levels of the p-PI3K (*p* < 0.01), PI3K (*p* < 0.05), p-Akt (*p* < 0.05), and Akt (*p* < 0.01) proteins were remarkably decreased compared with the Model group, while no significant differences were observed between the L-LVM and H-LVM groups.

Immunostaining was conducted to detect the expression of the PI3K and Akt proteins in the synovium of rats from different treatment groups, with the PI3K and Akt proteins appearing brown under the microscope (Figure 6B). Compared with the Control group, the synovial tissues of rats in the Model group exhibited a significant distribution of brown particles, along with evident changes in synovial tissue structure. Conversely, treatment with MTX and M-LVM significantly reduced the expression of the PI3K and Akt proteins in comparison to the Model group. In the L-LVM and H-LVM groups, a significant distribution of brown particles in the synovial tissues was observed, with a pathological status similar to that of the Model group.

### 2.6. Safety Evaluations

**Figure 7:**
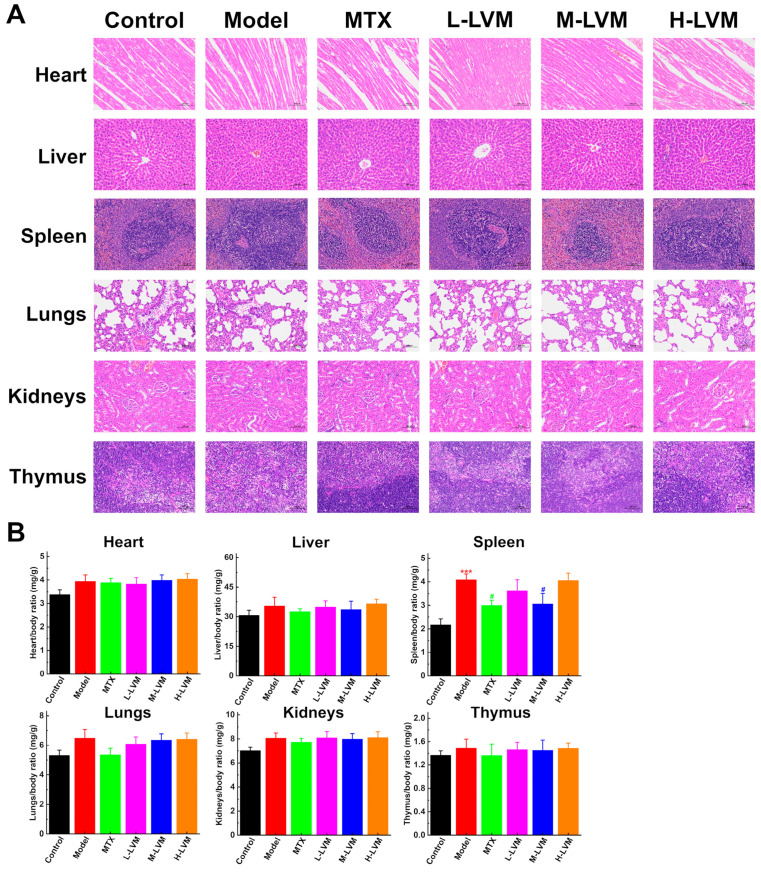
Evaluation of potential adverse effects of LVM in AIA rats. (**A**) The results of H&E staining (scale bar, 100 μm). (**B**) Organ coefficient of major organs. All data are shown as mean ± SEM. *** *p* < 0.001 when comparing with the Control group using an unpaired Student’s *t*-test; ^#^ *p* < 0.05 when comparing with the Model group using one-way ANOVA followed by Dunnett’s post hoc test. Definitions of abbreviations for each dosing group: Control, blank control group; Model, model group; MTX, positive drug group; L-LVM, 5 mg/kg LVM group; M-LVM, 15 mg/kg LVM group; H-LVM, 45 mg/kg LVM group. *n* = 6 in each group.

In Figure 7A, apart from the spleen, no substantial organic lesions were observed in the heart, liver, lungs, kidneys, or thymus tissues of the rats in each group. Additionally, the organ coefficients of these organs also showed few distinct differences compared with the Control group. However, as expected, the medullary regions of spleen tissues were significantly enlarged with enlarged germinal centers in both the Model and partial LVM-treated groups compared with the Control group, whereas this symptom was ameliorated in the MTX and M-LVM groups. This observation is further reflected in the organ coefficient results (Figure 7B), where the spleen coefficients of the Model group and partial LVM-treated groups showed a large increase relative to the Control group, while no significant difference was observed in the MTX and M-LVM groups, suggesting that the establishment of the AIA model may lead to changes in the normal physiological structure of the spleen tissue.

## 3. Discussion

RA is an autoimmune disease characterized by distinctive inflammation, exhibiting significant individual variability throughout its progression, often accompanied by multi-systemic involvement [41]. Although there is currently no cure for RA, early diagnosis, intervention, and treatment are crucial for controlling disease progression and alleviating symptoms [42]. Consequently, effective treatment strategies for RA have become a focal point of research. Animal models of RA can mimic the pathological processes of human RA, facilitating a deeper understanding of the pathophysiological changes and mechanisms underlying the disease [43]. This, in turn, may lead to the identification of novel therapeutic targets and the subsequent screening and evaluation of potential pharmacological agents. The AIA rat model shares many similarities with human RA, including genetic susceptibility, overactivation of cellular immunity, and similar joint damage [43]. The AIA model constructed in this study utilizes complete Freund’s adjuvant (CFA) or incomplete Freund’s adjuvant (IFA) for model induction. The pathogenesis of this model primarily involves the subcutaneous injection of CFA or IFA into the tail base, triggering immune responses by macrophages, T cells, and B cells to antigens and promoting the production of inflammatory cytokines such as IL-1β and TNF-α, resulting in swelling and pain in multiple periarticular tissues [44,45]. CFA primarily consists of inactivated *M. tuberculosis* mixed with mineral oil, which effectively activates the immune system due to its antigenic stimulatory substances. IFA, a variant of CFA, typically lacks bacterial components, retaining only mineral oil, thus inducing a lower degree of immune system stimulation, requiring multiple immunizations to induce the model. Consequently, our research group established the AIA model by subcutaneously injecting CFA at the base of the tail to replicate the pathogenesis and clinical manifestations of human RA for experimental investigation.

As a previously used clinical drug, LVM is administered for RA treatment in adults at doses of either 50 mg or 150 mg per day [46,47,48]. According to the practice guide for dose conversion between animals and humans [49,50,51], based on a clinical adult dosage of 150 mg per day, the calculated dosage for rats is 13.5 mg/kg/day. For ease of calculation, low (5 mg/kg), median (15 mg/kg), and high (45 mg/kg) treatment doses were established. The findings indicate that within both the Model group and three LVM treatment groups, one or two asymptomatic rats were observed attributed to individual variations and procedural factors. However, the MTX group and M-LVM group rats, which demonstrated therapeutical effect, showed mild inflammatory lesions in synovial and ankle joint tissues. Additionally, there were slight increases or decreases in the levels of pro-/anti-inflammatory cytokines in the serum compared with the Control group. Overall, compared with the Model, L-LVM, and H-LVM groups, treatment with M-LVM reduced the incidence rate, significantly alleviated foot swelling and inflammatory lesions in the synovium and ankle joints, and regulated pro-/anti-inflammatory cytokines in the serum to normal levels.

Histopathological examination of major organs revealed no significant organic changes or abnormal proliferation, except for the spleen. Treatment with MTX and M-LVM alleviated the abnormal changes in the spleen, suggesting that immune abnormalities induced by the model might lead to structural changes in spleen tissue. Serum biochemical tests showed no liver or kidney function abnormalities. However, CK levels, indicative of muscle damage, were elevated in the Model, L-LVM, and H-LVM groups, but significantly reduced after M-LVM and MTX treatment. RA patients have a higher incidence of abnormal glucose metabolism compared to the general population, possibly due to persistent systemic inflammation [52,53,54]. Our results show that M-LVM and MTX treatment normalized serum GLU levels. ALP, crucial for bone metabolism [55,56], can be affected by abnormal immune activity, leading to excessive bone formation and mineralization. In this study, M-LVM and MTX treatment reduced serum ALP levels to normal, suggesting a potential regulatory effect on bone metabolism in the RA context.

During the progression of RA, excessive immune activation leads to the accumulation of various immune cells in the synovium, resulting in the release of multiple cytokines, causing synovial inflammation and triggering joint swelling and cartilage destruction, exacerbating joint damage [57,58]. The PI3K/Akt signaling pathway is closely associated with RA, as it can stimulate the proliferation of fibroblast-like synoviocytes and induce synovial inflammation by promoting the release of inflammatory factors such as IL-1β, IL-6, IL-17, and TNF-α. Additionally, it is crucial for the proliferation and survival of immune cells such as T cells, B cells, and macrophages [59,60,61,62]. Western blotting and immunohistochemistry staining results demonstrate that the expression of PI3K- and Akt-related proteins in the synovial and ankle joint tissues of rats in the M-LVM group was significantly reduced, while no regulatory effect on the PI3K/Akt signaling pathway was observed after treatment with L-LVM and H-LVM.

In general, both the curative effect and mechanistic investigation results indicate the dual nature of LVM treatment. Neither low nor high doses demonstrated therapeutic effects; only within a specific dosage range did it show the ability to alleviate inflammatory symptoms, suggesting a seemingly contradictory immunomodulatory effect [63]. As reported in the study [64], LVM displayed immunomodulatory effects in a pneumonia rat model, with outcomes varying between pro-inflammatory and anti-inflammatory responses depending on the dosage, showing the close correlation between the pharmacological activity of LVM and its dosage. Moreover, hydrogen sulfide (H_2_S) serves as a crucial gasotransmitter in biological systems, exerting various physiological and protective effects. Optimal levels of H_2_S alleviate inflammatory reactions, while excessively high or low doses may have adverse effects, potentially exacerbating inflammation and tissue damage [65,66]. Consequently, our group hypothesized that the low dose of LVM might not effectively inhibit the PI3K/Akt signaling pathway to restore imbalanced immune function to normal levels, thus failing to exert anti-inflammatory effects. Conversely, the high dose of LVM might overly activate the PI3K/Akt signaling pathway, exacerbating immune function imbalance and worsening synovial and ankle joint inflammatory lesions. Only by intervening with the appropriate dose could the regulatory role of imbalanced immune function be realized, thereby suppressing inflammation and demonstrating therapeutic effects on RA.

## 4. Materials and Methods

### 4.1. Materials

LVM was provided by Nanjing Baijingyu (Nanjing, Jiangsu, China). NaCl, anhydrous ethanol, xylene, hydrochloric acid, and neutral gum were purchased from Sinopharm Chemical Reagent (Shanghai, China). MTX hydrate was provided by Macklin (Shanghai, China). Desiccated *M. tuberculosis* H37Ra was obtained from BD Difco (Sparks, MD, USA). Mineral oil was purchased from Sigma Aldrich (St. Louis, MO, USA). Rat IL-1β ELISA kit, rat TNF-α ELISA kit, and rat TGF-β1 ELISA kit were purchased from Multisciences (Hangzhou, Zhejiang, China). CREA kit, BUN kit, TP kit, ALB kit, AST kit, ALT kit, TBIL kit, ALP kit, TG kit, CHOL kit, GLU kit, and CK kit were purchased from Hzymes Biotech (Wuhan, Hubei, China). Phosphatase inhibitor cocktail was purchased from MedChemExpress (Monmouth Junction, NJ, USA). Phenylmethylsulfonyl fluoride, primary antibody dilution buffer, stripping and reprobing Western blotting membranes, and radio-immunoprecipitation assay (RIPA) buffer were provided by Guangzhou Meilunbio (Guangzhou, Guangdong, China). Bicinchoninic acid assay (BCA) protein assay kit was purchased from Solarbio (Beijing, China). Phosphate-buffered saline (PBS), hematoxylin and eosin staining kit, bovine serum albumin (BSA), antigen retrieval solution, tissue fixatives, rabbit serum and immunohistochemistry kit, protein loading buffer, pre-stained protein marker, electrophoresis buffer, transfer buffer, tris-buffered saline (TBS), protein-free blocking solution, and anti-GAPDH antibody were provided by Wuhan Saiweier Biotechnology (Wuhan, Hubei, China). PAGE gel fast preparation kit was provided by Shanghai Epizyme Biomedical Technology (Shanghai, China). Tween-20 was purchased from Shanghai Aladdin Biochemical Technology (Shanghai, China). Hydrophobic polyvinylidene fluoride (PVDF) transfer membrane was purchased from Merck (Darmstadt, Hessen Land, Germany). HRP-labeled goat anti-rabbit IgG (H+L) and BeyoECL star were provided by Beyotime Biotechnology (Shanghai, China). Primary antibodies against PI3K, p-Akt, and Akt were purchased from Cell Signaling Technology (Danvers, MA, USA). Primary antibody against p-PI3K was purchased from Biosynthesis Biotechnology (Beijing, China).

### 4.2. Animals

Sprague–Dawley (SD) rats (male, 4–5 weeks old, weighing 150 ± 20 g) were procured from the Shanghai Slac Laboratory Animal Company (License no. SCXK 2022-0004, Shanghai, China) and housed in a standard laboratory animal facility at a temperature of 26 ± 2 °C with a 12 h light/12 h dark cycle. Rats had access to water and food ad libitum throughout the duration of the experiment. Prior to the commencement of the study, the animals underwent a 4-day acclimation period. All surgical procedures were performed under pentobarbital anesthesia (2%, dissolved in saline, 3 mL/kg), and euthanasia was carried out using CO_2_ in a sealed container. This study was approved by the Animal Ethics Committee of Fujian Medical University (Approval No: FJMU IACUC 2023-0157).

### 4.3. AIA Rat Model

**Figure 8:**
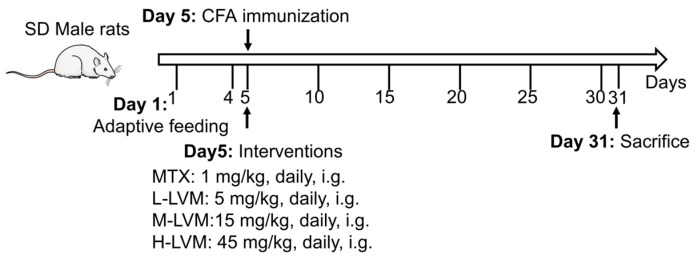
The experimental schematic.

The experimental procedure is illustrated in Figure 8. The AIA rat model was constructed by a single subcutaneous injection at the base of the tail of *M. tuberculosis* H37Ra suspended in mineral oil (10 mg/mL, 0.1 mL per rat) on day 5 [67]. Rats were divided into the following 6 groups: Control group (subcutaneously injected with saline once and oral gavage administrated with PBS daily, *n* = 6), Model group (immunized and oral gavage administrated with PBS daily, *n* = 6), MTX group (immunized and oral gavage administrated with MTX daily, 1 mg/kg, *n* = 6), L-LVM group (immunized and oral gavage administrated with LVM daily, 5 mg/kg, *n* = 6), M-LVM group (immunized and oral gavage administrated with LVM daily, 15 mg/kg, *n* = 6), and H-LVM group (immunized and oral gavage administrated with LVM daily, 45 mg/kg, *n* = 6). From the first day of modeling, MTX and LVM treatments were initiated, with the entire treatment period lasting 25 days. MTX and LVM were dissolved in PBS.

### 4.4. Arthritis Index Scoring

The arthritis index was recorded at the 0th, 5th, 10th, 15th, 20th, and 25th days post-modeling. The scoring criteria were as follows: grade 0, normal, no redness or swelling; grade 1, redness or mild swelling limited to the ankle and metatarsophalangeal joints; grade 2, redness and moderate swelling from the ankle to the midfoot; grade 3, redness and severe swelling from the ankle to the tarsometatarsal joints; grade 4, ankylosis deformity including the ankle and swelling of the footpads [68]. The cumulative score of the four limb joints represented the arthritis index score for each rat, with a maximum score of 16 points.

### 4.5. Foot Pad Thickness and Paw Volume Analysis

Prior to using the instrument, zero calibration was performed. Rats were immobilized, and a line was drawn at the same position on the right hind paw of each rat for marking [69]. Subsequently, the thickness of the paw pad at the marked position was measured using an electronic digital caliper (Vogel Germany, Kevelaer, Nordrhein-Westfalen, Germany), while the right hind paw was gradually submerged into a measuring cup filled with water to determine the drainage volume using a plethysmometer (YAN YI-TECH, Jinan, Shandong, China). Measurements were conducted at the 0th, 5th, 10th, 15th, 20th, and 25th days post-modeling, with three parallel measurements taken each time, and the average value was calculated.

### 4.6. Micro-CT Analysis

After euthanizing the rats, hind limbs were obtained and scanned using micro-CT (Bruker, Billerica, MA, USA) to acquire flat and 3D reconstructed images of the rat ankle joint and foot [70]. The parameters were set as follows: voltage 60 kV, current 166 μA, and exposure time 1399 ms.

### 4.7. Histopathology

According to the methods described in the study, rat tissues were rapidly excised and immediately immersed in 4% paraformaldehyde for fixation, ensuring the preservation of cellular architecture and biochemical integrity [71]. Following fixation, the specimens underwent a dehydration process utilizing a series of increasingly concentrated alcohol solutions. Subsequently, the dehydrated tissues were treated with a clearing agent and infiltrated with paraffin wax. Thin sections of the paraffin-embedded tissues were obtained using a microtome. These sections were then subjected to H&E staining to delineate specific cellular components, with hematoxylin imparting a blue coloration to cell nuclei and eosin staining the cytoplasm and extracellular matrix a pink hue. Upon completion of the staining process, the cellular morphology, tissue architecture, and any pathological alterations were meticulously examined under a microscope. Additionally, due to the presence of bone structures in the ankle joint, decalcification treatment was performed after fixation.

### 4.8. ELISA

The levels of IL-1β, TNF-α, and TGF-β in rat serum were measured using rat-specific ELISA kits. The quantification of three cytokines was conducted using a double antibody sandwich ELISA technology. A specific capture antibody was pre-coated onto a high-affinity ELISA plate. Subsequently, a standard, test sample, and biotinylated detection antibody were added to the wells of the ELISA plate. Following incubation, the analyte in the sample bound to both the capture and detection antibodies. After washing to remove unbound substances, horseradish peroxidase-conjugated streptavidin was added. Another washing step was performed before introducing the chromogenic substrate, tetramethylbenzidine (TMB), while avoiding exposure to light to facilitate color development. The intensity of the resulting color is directly proportional to the concentration of the target analyte in the sample. A termination solution was then added to stop the reaction, and the optical density (OD) was calibrated by subtracting the measured value at 570 nm from that at 450 nm. Finally, a standard curve was generated through regression analysis, allowing for the calculation of concentrations of IL-1β, TNF-α, and TGF-β in the test samples by multiplying the obtained values by the corresponding dilution factors.

### 4.9. Serum Biochemical Detection

Biochemical tests can reflect whether various indicators related to liver and kidney function, blood lipids, and blood glucose are normal in organisms. Therefore, a fully automated biochemical analyzer (Beckman, Brea, CA, USA) was employed to measure the levels of CREA, BUN, TP, ALB, AST, ALT, TBIL, ALP, TG, CHOL, GLU, and CK in rat serum. In short, following the meticulous collection of blood samples, they were subjected to centrifugation for the separation of serum from cellular components. Prior to the initiation of the assays, all requisite reagents were prepared according to the manufacturer’s instructions and calibration of the automated analyzer was performed using a series of standard solutions. Once calibration was completed, serum samples were placed into the sample tray of the automated analyzer. The biochemical assays were executed by the analyzer through a series of programmed steps, including dispensing, reagent addition, and incubation. After the incubation period, the OD of each reaction mixture was measured using photometric methods. The resulting optical density values were processed through integrated software algorithms that interpolated the results against established calibration curves. Finally, the concentrations of the biochemical markers were calculated by the analyzer and any necessary dilution factors were applied to yield the final results.

### 4.10. Western Blotting

Rat tissues were weighed, and a mixed lysis buffer consisting of RIPA, protease inhibitors, and phosphatase inhibitors was added to prevent protein degradation. Following grinding, lysis, and centrifugation, the protein concentrations of each sample were determined using the BCA and denaturation of the proteins was performed [72]. Subsequently, the samples were loaded into the wells of an SDS-PAGE gel and electrophoresis was conducted to separate the proteins based on their molecular weight. The gel was run at a constant voltage until the dye front migrated an appropriate distance. After electrophoresis, proteins were transferred from the gel to a PVDF membrane using a TransBlot system (Bio-Rad, Hercules, CA, USA). To minimize non-specific binding of antibodies, the membrane was incubated with non-fat dry milk for a duration of 1–2 h at room temperature or overnight at 4 °C. The membrane was then incubated with a primary antibody specific to the target protein overnight at 4 °C, depending on the antibody and the desired sensitivity. Following this incubation, the membrane was washed several times with a wash buffer containing a low concentration of detergent (Tween-20) to remove any unbound antibodies. After washing, the membrane was incubated with horseradish peroxidase-conjugated secondary antibody. In accordance with the previous step, the membrane was subsequently washed again to eliminate any unbound secondary antibodies. For antibody stripping, the membrane was incubated in a stripping buffer containing phenylmethylsulfonyl fluoride for a specified period of 15–30 min at 50 °C. Following stripping, the membrane was thoroughly washed to remove any residual stripping buffer before being reblocked and reprobed with a new primary antibody. Finally, the blots were visualized using BeyoECL Star and captured with a Bio-Rad ChemiDoc MP imaging system (Bio-Rad, Hercules, CA, USA). Quantitative data were obtained using ImageJ software (Version 1.8.0.112).

### 4.11. Immunohistochemistry Assay

After tissue preparation, fixation, embedding, sectioning, deparaffinization, and rehydration, the slides were placed in a retrieval buffer (citrate buffer) and heated in a microwave or a water bath for 20 to 30 min. The slides were then allowed to cool to room temperature, followed by washing in PBS to remove the retrieval buffer. The sections were incubated with a blocking solution (non-fat dry milk) for 30 to 60 min at room temperature to minimize non-specific binding of antibodies. The primary antibody was diluted in an appropriate dilution buffer and applied to the sections. Depending on the specificity and sensitivity requirements of the antibody, the sections were incubated overnight at 4 °C or for 1 to 2 h at room temperature. After incubation, the sections were washed several times in PBS (typically 3 to 5 washes of 5 min each) to remove unbound primary antibodies and an appropriate secondary antibody was then applied. The sections were washed again in PBS to eliminate any unbound secondary antibodies. Subsequently, the sections were incubated with a substrate solution that reacted with the enzyme conjugated to the secondary antibody, resulting in the formation of a colored precipitate at the site of the target antigen. The sections were counterstained with hematoxylin for 1 to 5 min to visualize the cellular architecture. The sections were dehydrated through increasing concentrations of ethanol (50%, 70%, 95%, and 100%) followed by clearing in xylene and then covered with a glass coverslip for microscopy. Finally, the sections were examined under a light microscope and images were captured for subsequent analysis.

### 4.12. Statistical Analysis

Quantitative data were expressed as the mean ± standard error of the mean (SEM). All analyses were conducted using GraphPad Prism version 8.02 (GraphPad Software Inc., San Diego, CA, USA). Continuous variables were compared using appropriate *t*-tests, with normality assessed via the Shapiro–Wilk test. Statistical significance was defined as follows: * *p* < 0.05, ** *p* < 0.01, and *** *p* < 0.001 when comparing to the Control group using an unpaired Student’s *t*-test; ^#^ *p* < 0.05, ^##^ *p* < 0.01, and ^###^ *p* < 0.001 when comparing to the Model group using one-way ANOVA followed by Dunnett’s post hoc test.

## 5. Conclusions

In summary, this study demonstrates that treatment with M-LVM effectively alleviated foot symptoms in AIA rats, including reduced foot swelling, improved synovial and ankle joint pathologies, and normalized inflammatory markers and serum biochemistry. The therapeutic mechanism in the M-LVM group appears to involve modulation of the PI3K/Akt signaling pathway, as evidenced by decreased expression of related proteins in synovial and ankle joint tissues. Our findings suggest that LVM, at an optimal dosage, shows promise as a potential therapeutic agent for RA, with elucidated underlying mechanisms.

## Data Availability

Data are contained within the article.
